# Increasing ATP conservation in maltose consuming yeast, a challenge for industrial organic acid production in non-aerated reactors

**DOI:** 10.1186/1753-6561-8-S4-P185

**Published:** 2014-10-01

**Authors:** Stefan de Kok, Wesley Leoricy Marques, Robert Mans, Duygu Yilmaz, Erwin Suir, Jack T Pronk, Andreas K Gombert, Jean-Marc Daran, Antonius JA van Maris

**Affiliations:** 1 Department of Biotechnology, Delft University of Technology and Kluyver Centre for Genomics of Industrial Fermentation, Julianalaan 67, 2628 BC Delft, The Netherlands; 2 Department of Chemical Engineering, University of São Paulo, São Paulo, Brazil; 3 Faculty of Food Engineering, University of Campinas, São Paulo, São Paulo, Brazil

## 

Anaerobic fermentation processes are economically attractive for industry, as costs for aeration and stirring are greatly reduced. An example of such a process is (bio)ethanol production by *Saccharomyces cerevisiae*, in which a low but positive ATP gain leads to elevated product yields. However, for other fermentative pathways, such as in an engineered homolactic strain of *S. cerevisiae*, the net ATP gain for the fermentation of glucose to lactate is null due to the requirement of ATP for product export. Therefore, increasing the conservation of ATP is of major importance for such 'zero-ATP pathways'.

One opportunity to increase ATP conservation arises when disaccharides are used as a substrate. In many industrial microorganisms, disaccharides are cleaved by hydrolysis, which results in the dissipation of energy that is available in this cleaving reaction. However, phosphorolytic cleavage could be used to replace the hydrolysis of disaccharides, thereby increasing the ATP yield.

In this study, growth of *S. cerevisiae *on maltose was used as a model. All known native maltose metabolism genes were removed and replaced by a maltose phosphorylase (*Lactobacillus sanfranciscensis*) and a single overexpressed copy of the native *MAL11 *maltose transporter. Because maltose phosphorylase cleaves maltose into glucose and β-glucose-1-phosphate, additionally a β-phosphoglucomutase (*Lactococcus lactis*) was co-expressed in this strain.

Anaerobic maltose-limited chemostat cultures showed that replacement of maltose hydrolysis by phosphorolysis increased the biomass yield of the mutant strain by 26% over the wild type strain, demonstrating the potential of phosphorolysis to improve ATP conservation of disaccharide metabolism in industrial microorganisms [1].
